# Uncoating Mechanism of *Carnation Mottle Virus* Revealed by Cryo-EM Single Particle Analysis

**DOI:** 10.1038/srep14825

**Published:** 2015-10-07

**Authors:** Chun-Yan Wang, Qin-Fen Zhang, Yuan-Zhu Gao, Li Xie, Hong-Mei Li, Jian Hong, Chuan-Xi Zhang

**Affiliations:** 1 College of Agriculture and Biotechnology, Zhejiang University, Hangzhou 310058, China; 2 State Key Laboratory of Biocontrol, School of Life Sciences, Sun Yat-sen University, Guangzhou 510275, China; 3 Institute of Virology and Biotechnology, Zhejiang Academy of Agricultural Sciences, Hangzhou 310021, China

## Abstract

Genome uncoating is a prerequisite for the successful infection of plant viruses in host plants. Thus far, little is known about the genome uncoating of the *Carnation mottle virus* (CarMV). Here, we obtained two reconstructions of CarMV at pH7 in the presence (Ca-pH7) and absence (EDTA-pH7) of calcium ions by Cryo-EM single particle analysis, which achieved 6.4 Å and 8 Å resolutions respectively. Our results showed that chelation of the calcium ions under EDTA-pH7 resulted in reduced interaction between the subunits near the center of the asymmetric unit but not overall size change of the viral particles, which indicated that the role of the calcium ions in CarMV was not predominantly for the structural preservation. Part of the genomic RNA closest to the capsid was found to be located near the center of the asymmetric unit, which might result from the interaction between genomic RNA and Lys194 residues. Together with the electrostatic potential analysis on the inner surface of the asymmetric unit, the reduced interaction near the center of the asymmetric unit under EDTA-pH7 suggested that the genome release of CarMV might be realized through the center of the asymmetric unit.

*Tombusviridae* viruses are positive-strand RNA viruses with icosahedral symmetry, of which many representative structures in the genera have been determined, including *Carnation mottle virus* (CarMV)^1^, *Tobacco necrosis* virus A (TNV-A)^2^, *Panicum mosaic virus* (PMV)^3^ and *Tomato bushy stunt virus* (TBSV)^4^, as well as other non-representative members such as *Turnip crinkle virus* (TCV)^5^, *Melon necrotic spot virus* (MNSV)^6^, *Cowpea mottle virus* (CPMoV)^7^ and *Cucumber necrosis virus* (CNV)^8^. Based on the surface features, the viruses in the family *Tombusviridae* can, thus far, be divided into two groups: TBSV-like and TNV-like viruses. TBSV-like viruses possess obvious protruding pentamers and hexamers on the surface, while TNV-like viruses present a rather smooth surface. CarMV, TBSV, TCV, MNSV, CPMoV and CNV are all grouped into TBSV-like viruses^1^,^4^,^5^,^6^,^7^,^8^. The calcium ions are located in the three subunit interfaces within one asymmetric unit of the TBSV-like viruses^4^,^5^,^6^,^7^,^8^ except for CarMV, in which only one calcium ion is found in the interface of the B subunit and C subunit^1^. In the case of TBSV-like viruses, an expanded state resulting from the chelation of the calcium ions under certain conditions has been found, which is considered to be one intermediate of viral disassembly^9^,^10^. The structure of TBSV at 8 Å resolution demonstrates that the conformations of viral subunits in the expanded state are only locally changed, with the folded domains well preserved^9^. Moreover, the expanded states are also found for the viruses that do not belong to the family *Tombusviridae*, such as the *Southern bean mosaic virus* (SBMV)^11^ and *Cowpea chlorotic mottle virus* (CCMV)^12^. The common feature of the viruses with expanded state mentioned above is that the calcium ions are located in the three subunit interfaces within one asymmetric unit.

CarMV is the representative member of the genus *Carmovirus* in the family *Tombusviridae*, the capsid of which consists of 180 capsid proteins with identical sequences^1^,^13^. The structure of the CarMV capsid has been determined by X-ray diffraction analysis, in which the calcium ions are only found in the interface of the B subunit and C subunit within one asymmetric unit^1^. However, the role of the calcium ion in the structure of the CarMV has not been determined, and the genome uncoating mechanism of CarMV remains unknown. Here, we obtained the native structures of CarMV under two conditions, pH7 in the presence of calcium ions (Ca-pH7) and absence of calcium ions (EDTA-pH7) by Cryo-EM single particle analysis and revealed structural differences of the subunit interfaces within one asymmetric unit in the two reconstructions. Together with the electrostatic potential analysis on the inner surface, a model of CarMV genome uncoating was proposed.

## Results

### Cryo-EM reconstructions and resolution assessment

**Figure 1:**
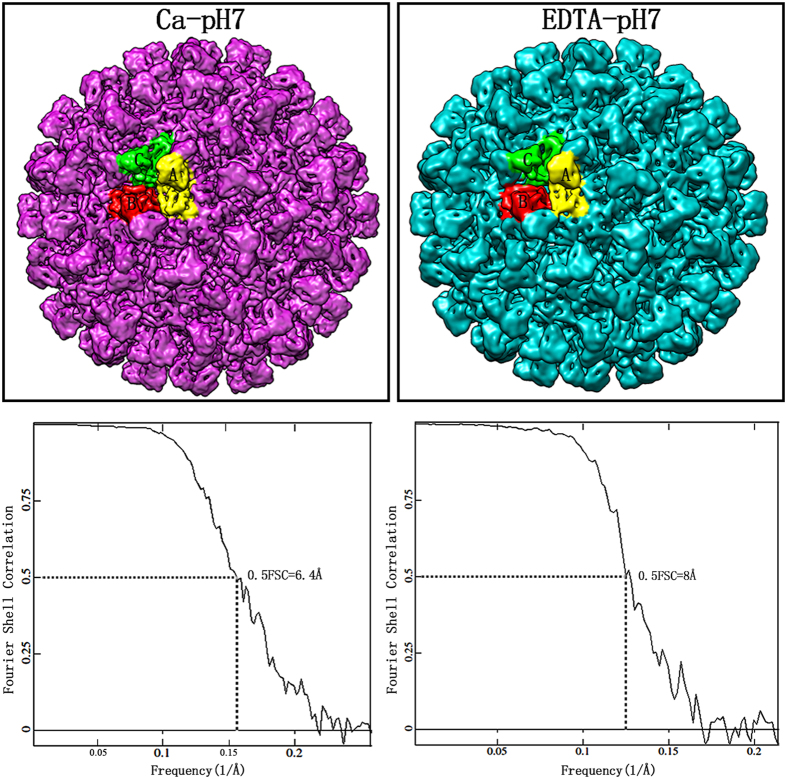
Three-dimensional reconstructions and resolution assessment. The density maps of CarMV under Ca-pH7 (purple) and EDTA-pH7 (cyan) with three subunits in one asymmetric unit presented in different colors: **A** subunit (yellow), **B** subunit (orange red) and **C** subunit (green). The same color scheme is applied throughout this paper. The density maps are visualized with the contour level set at 2.5 in Chimera. The FSC curves between the even-odd reconstructions are computed, indicating that the resolutions of the final density maps are estimated at 6.4 Å (Ca-pH7) and 8 Å (EDTA-pH7), respectively, using the 0.5 FSC criterion.

**Figure 2:**
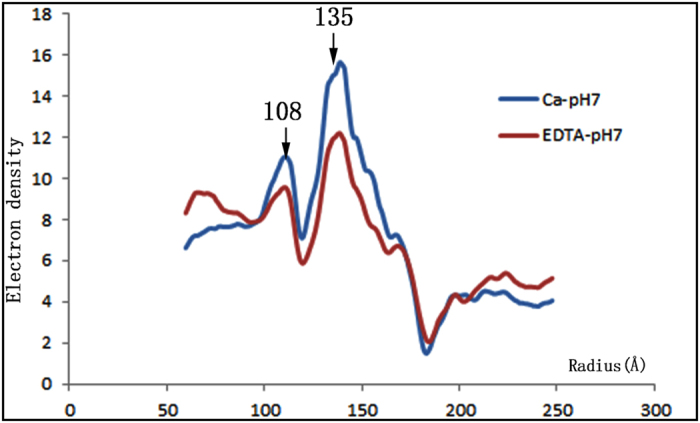
Density distribution plots. Two density peaks are located at radii of ~108 Å and ~135 Å in the density distribution plots, under Ca-pH7 (blue) and EDTA-pH7 (red), with the x-axis ranging from 50 Å to 250 Å, and are labeled and indicated with arrows.

The three-dimensional structures of CarMV were reconstructed from virus samples under Ca-pH7 and EDTA-pH7. In total, ~18, 800 particles are used for the final reconstruction under Ca-pH7 and ~10, 400 particles for the final reconstruction under EDTA-pH7. The Cryo-EM results showed that CarMV belongs to the typical T = 3 icosahedral viruses with crown-like pentamers and hexamers on the surface (Fig. 1). The basic building block of the capsid was the asymmetric unit, consisting of three chemically identical subunits. Five copies of the A subunit were packed around the five-fold axis, and three copies of the B subunit and C subunit were packed around the three-fold axis, alternating (Fig. 1). Due to the limited number of particles, we did not divide the total particles into two parts and perform independent reconstructions to estimate the resolution^14^. Instead, the same initial model was utilized to determine the orientations of all the particles and two semi-independent models were obtained. The FSC curve between the two semi-independent reconstructions was computed, and the resolutions of the two reconstructions were assessed at 6.4 Å (Ca-pH7) and 8 Å (EDTA-pH7), respectively, using the 0.5 FSC criterion^15^ (Fig. 1). The density distribution plots of the two reconstructions showed that two obvious density peaks were located at radii of ~108 Å and ~135 Å respectively (Fig. 2). The highest density peak at the radius of ~135 Å was contributed by the capsid, while the second-highest density peak at the radius of ~108 Å was contributed by the genomic RNA. Based on the density distribution plots, the overall size of the two reconstructions showed no obvious difference, sharing similar peaks and wave troughs (Fig. 2).

### Interactions between the subunits within one asymmetric unit

**Figure 3:**
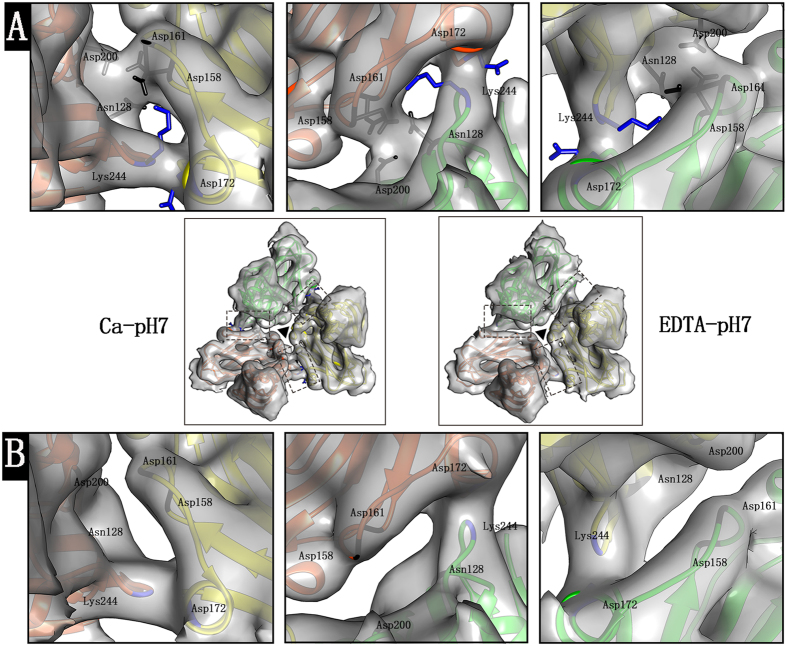
Interactions between the subunits within one asymmetric unit. **(A)** Zoom-in view of the linking densities boxed in the subunit interfaces within one asymmetric unit under Ca-pH7 located in the middle panel between panel (**A**) and panel (**B**) (left). One linking density is surrounded by the residues Asn128, Asp158, Asp161 and Asp200, colored in black, and the other linking density is surrounded by the residues Lys244 and Asp172, colored in blue. **(B)** Zoom-in view of the linking densities boxed in the subunit interfaces within one asymmetric unit under EDTA-pH7 located in the middle panel between panel (**A**) and panel (**B**) (right). The linking density is surrounded by the residues Lys244 and Asp172. The centers of the asymmetric units are marked with black triangles. The density maps are normalized and visualized with the contour level set at 2.5 in Chimera.

To compare the interaction between the subunits within one asymmetric unit, the density maps under Ca-pH7 and EDTA-pH7 were low-pass filtered at the same resolution of 8 Å, which was the lower resolution of the two reconstructions. The linking densities between the subunits within one asymmetric unit under Ca-pH7 and EDTA-pH7 were visualized (Fig. 3). In the reconstruction under Ca-pH7, two linking densities were found to be located in each subunit interface within one asymmetric unit, while only one linking density was found under EDTA-pH7 (Fig. 3A,B). The model of the CarMV (PDB ID: 1OPO) from X-ray diffraction analysis was docked in the density map under Ca-pH7, which showed that the linking density located closer to the center of the asymmetric unit was contributed by the residues Asn128, Asp158, Asp161 and Asp200 (Fig. 3A) and the other, located further from the center of the asymmetric unit, was contributed by the residues Lys244 and Asp172 (Fig. 3A). The flexible fitting result of the CarMV X-ray structure (PDB ID: 1OPO) in the density map under EDTA-pH7 showed that the Cα positions οf the residues Asn128, Asp158, Asp161, Asp200, Lys244 and Asp172 were not changed, which was docked in the density map under EDTA-pH7 (Fig. 3B). However, the side-chain orientations were hard to determine at the obtained resolution. The fitting result demonstrated that the linking density contributed by the residues Lys244 and Asp172 under Ca-pH7 remained in the reconstruction under EDTA-pH7, while the linking density surrounded by the residues Asn128, Asp158, Asp161 and Asp200 under Ca-pH7 was absent under EDTA-pH7 (Fig. 3B).

### Interactions between the genomic RNA and capsid

**Figure 4:**
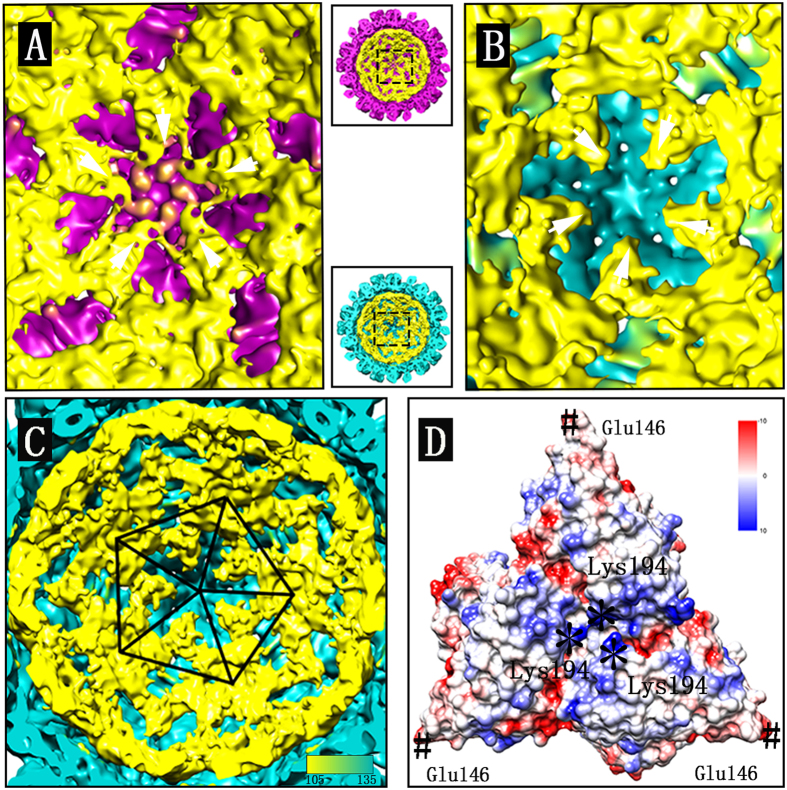
Inside views of the reconstructions. (**A**) Zoom-in view of the pentamer inside the reconstruction under Ca-pH7 in the middle panel between panel (**A**) and panel **(B)** (up). **(B)** Zoom-in view of the pentamer inside the reconstruction under EDTA-pH7 in the middle panel between panel (**A**) and panel (**B**) (low). **(C)** One pentamer composed of five asymmetric units is outlined inside the reconstruction under EDTA-pH7. **(D)** The asymmetric unit of the X-ray structure colored by Coulombic electrostatic potential shows that three Lys194 residues are located near the center of the asymmetric unit on the inner surface as marked with *, and three Glu146 residues are located at the vertices of the triangular asymmetric unit as marked with #. The genomic RNA densities close to the capsid are colored in yellow. All the density maps shown are low-pass filtered at 8 Å resolution and normalized before visualization with the contour level set at 1.8 in Chimera.

As a result of the limitations of the reconstruction algorithm, the structure of the genomic RNA inside the capsid could not be fully resolved. However, the electron density distribution closest to the outer shell might indicate potential interaction between the genomic RNA and capsid. In our study, the electron density distribution closest to the inner surface of the capsid was visualized, part of which was found to be located near the center of the asymmetric unit (Fig. 4A–C). Extra interaction between the genomic RNA and capsid was observed near the center of the pentamers in the reconstructions under Ca-pH7 but not under EDTA-pH7 (Fig. 4A,B). The density distribution of the genomic RNA closest to the capsid was located near the radius of ~108 Å, which was exemplified from the inside view of the reconstruction under EDTA-pH7 (Fig. 4C). To better visualize the distribution of the genomic RNA closest to the capsid, the triangular asymmetric units packed along the five-fold axis were outlined with one pentamer composed of five triangles (Fig. 4C). The X-ray structure of CarMV (PDB ID: 1OPO) suggests that three Lys194 residues are located near the center of the asymmetric unit on the inner surface, which contribute to the positive charge at the corresponding location (Fig. 4D), while three Glu146 residues are located at the vertices of the triangular asymmetric unit (Fig. 4D), resulting in a negative charge at the five-fold axis and three-fold axis.

## Discussion

The difference between the reconstructions under Ca-pH7 and EDTA-pH7 predominantly resulted from the influence of EDTA on the structure of CarMV (Fig. 3A,B). In our results, two linking densities were found to be located in the subunit interfaces within one asymmetric unit under Ca-pH7 (Fig. 3A), while only one linking density was observed in the corresponding interfaces under EDTA-pH7 (Fig. 3B). The addition of EDTA might result in chelation of the calcium ions from the subunit interfaces and then led to reduction of the interactions within one asymmetric unit, which is consistent with our finding that the linking density surrounded by the residues Asn128, Asp158, Asp161 and Asp200 is absent under EDTA-pH7. The flexible fitting result showed that the Cα positions of the residues Asn128, Asp158, Asp161 and Asp200 were not changed under ETDA-pH7. Thus, it is speculated that removal of the calcium ions might result in changes to the side-chain orientations of these residues but not the Cα positions, as reflected by the absence of the linking density in the interface of the B subunit and C subunit under EDTA-pH7. The presence of the linking densities surrounded by the residues Asn128, Asp158, Asp161 and Asp200 in the B-C subunit interface under Ca-pH7 was understandable, due to the finding that only one calcium ion was located in the B-C subunit interface within one asymmetric unit based on the X-ray structure of CarMV (PDB: 1OPO)^1^. However, the presence of the densities located in the other two interfaces under Ca-pH7 requires further analysis^1^. One possibility is that the calcium ions might also exist in the other two interfaces under Ca-pH7, as the addition of EDTA has resulted in the absence of the linking densities in the three interfaces of the subunits within one asymmetric unit under EDTA-pH7 (Fig. 3B).

Early biochemical experimentation has suggested that the assembly of CarMV particles does not require the presence of calcium ions^1^. In this study, we found that absence of the calcium ions in the reconstruction under EDTA-pH7 did not result in the overall size change of the viral particles, which would further support the idea that the role of the calcium ions in CarMV is not primarily for the structure preservation. It seems that the calcium ions serve different functions in different viruses: in some viruses, they are critical to viral assembly^16^,^17^,^18^; while in some other viruses, they are more closely related to viral stability and infectivity^18^,^19^,^20^. The mutations to the calcium-binding sites in the *Flock house virus* (FHV) have been found to result in reduced viral stability and infectivity without changing the overall architecture of the capsid^20^, which serves as a good example that the calcium ions are related to viral infectivity. Similarly, we found that chelation of the calcium ions from the CarMV also reduced the subunit interactions within one asymmetric unit. Thus, the stability of the CarMV is speculated to be more or less reduced, and the infectivity of the virus was reduced as well. However, this conclusion is speculative, which still requires more biological evidence for further verification.

Genome release is a prerequisite for the successful infection of the viruses in hosts, allowing the genomic RNA to function as the template for RNA replication and protein translation, which are then used for the viral assembly^21^. The intracellular environment is generally not very harsh, which might not result in the collapse of viral particles. Thus, some specific mechanism might exist to regulate the genomic RNA release from the capsid. In the case of TCV, the expanded state with slits in the center of the asymmetric unit is considered to be an intermediate of viral genome uncoating^10^. Our findings showed that chelation of the calcium ions from CarMV under EDTA-pH7 reduced the interaction near the center of the asymmetric unit, which is speculated to be favorable to the exit of the genome, as the density of the genomic RNA might be located therein through interaction with the Lys194 residues. At the same time, the Glu146 residues located at the vertices of the triangular asymmetric unit form negatively charged centers at five-fold axis and three-fold axis, which might serve as a barrier against the leakage of the genomic RNA from the centers of pentamers and hexamers. Extra interaction between the genomic RNA and capsid was also found around the five-fold axis under Ca-pH7 but not under EDTA-pH7 (Fig. 4A,B), which indicated that the genomic RNA was more inclined to approach the center of the asymmetric unit under EDTA-pH7. Overall, the center of the asymmetric unit on the inner surface is likely to become the potential target of the genomic RNA. The model of the CarMV genome uncoating could be hypothesized as follows: the genomic RNA interacts with the capsid protein near the center of the asymmetric unit through interaction with the positively charged residue Lys194; chelation of calcium ions results in repulsion between the residues in the subunit interfaces within one asymmetric unit and then reduction of the interaction near the center of the asymmetric unit. The reduced interaction near the center of the asymmetric unit further facilitates the exit of the genomic RNA from the capsid; at the same time, the Glu146 residues form negative charges at the five-fold axis and three-fold axis, serving as a barrier against the leakage of the genomic RNA from the centers of the pentamers and hexamers. Once the genomic RNA passes the center of the asymmetric unit, these negatively charged residues contributing to the calcium ion binding locations might in turn function to facilitate the continuous transport of the genomic RNA.

## Material and Methods

### Purification and isolation of CarMV particles

The virus was isolated and purified from commercial stocks of carnation infected with CarMV. The sample was ground thoroughly in liquid nitrogen and then dissolved in 0.1 M phosphate buffer containing 0.1 M calcium chloride (pH7). After removing the impurities by centrifugation (12,000 g for 20 min at 4 °C), a mixture of equal volumes of chloroform and butanol was added to the supernatant at 1: 4 volume and mixed for 20 min at room temperature until the stratification disappeared. The mixture was then centrifuged (12,000  g for 20 min at 4°C), resulting in the butanol phase containing virus particles in the upper layer and the chloroform phase in the lower layer. The virus particles were then precipitated by ultracentrifuge (130,000 g for 2 h at 4 °C) (Beckman, USA). The resulting precipitation was resuspended and then centrifuged to remove the impurities (12,000 g for 20 min at 4 °C). The final buffer conditions were 0.01 M phosphate buffer pH7 with 0.01 M CaCl_2_ (Ca-pH7) and 0.01 M phosphate buffer pH7 with 0.01 M EDTA (EDTA-pH7).

### Cryo-EM sample preparation and data collection

A 3 μl sample was applied to 400 mesh Quantifoil R1.2/1.3 grids (Quantifoil, Germany) with freshly made, thin continuous carbon film, blotted for 3 s in the chamber of the Vitrobot IV at 100% humidity (FEI, USA) and then flash-frozen in liquid ethane. The EM data for the sample with the presence of calcium ions were collected on an FEI Titan Krios at 300 kV. The total dose used for each exposure was 20 electrons/Å^2^. The images were recorded on a Gatan UltraScan4000 (model 895) 16-megapixel CCD camera and the final pixel size was 1.196 Å/pixel. The defocus values ranged from −0.9~−3 μm. The EM data for the sample with the presence of EDTA were collected on an FEI Tecnai G^2^ TF20 at 200 kV. The images were recorded on an Eagle (FEI, USA) 4 k × 4 k CCD camera and the final pixel size was 1.35 Å/pixel.

### Image processing and Cryo-EM reconstruction

Particles were interactively selected from the cryo-EM images using the *e2boxer*.py program in EMAN2^22^. The contrast transfer function correction was performed using the *ctfit* in EMAN1.9 23. A starting model was generated using the *starticos* in EMAN1.9 23. The initial model was obtained from a small set of particles and then refined to the converged density map using the whole set of particles. The final density map was sharpened by applying B-factor using the *proc3d* in EMAN1.9 23. The resolution was assessed using the *eotest* program in EMAN1.9 23. The reconstructions under Ca-pH7 and EDTA-pH7 were performed using the same procedures.

### Segmentation, docking and visualization

The X-ray structure of CarMV (PDB ID: 1OPO) was downloaded and docked in the density map under Ca-pH7 using the “fit into map” module in Chimera^24^. The coloring of the subunits within one asymmetric unit was performed using the “color zone” module^24^. The asymmetric unit under EDTA-pH7 was segmented with the X-ray model as a track and the obtained density was fitted with the X-ray structure of CarMV to perform the flexible fitting procedure using Direx, which was available from the website https://simtk.org/home/direx/. The generated model was then docked in the density map of the asymmetric unit under EDTA-pH7 for further analysis on the linking densities in the subunit interfaces within one asymmetric unit. All the densities were normalized and visualized in Chimera^24^.

## Additional Information

**Accession numbers**: Cryo-EM density maps have been deposited in the Electron Microscopy Data Bank with the accession numbers EMD-6468 (Ca-pH7) and EMD-6469 (EDTA-pH7).

**How to cite this article**: Wang, C.Y. *et al*. Uncoating Mechanism of *Carnation Mottle Virus* Revealed by Cryo-EM Single Particle Analysis. *Sci. Rep*. **5**, 14825; doi: 10.1038/srep14825 (2015).
